# A systematic review of approaches to improve medication adherence in homeless adults with psychiatric disorders

**DOI:** 10.3389/fpsyt.2023.1339801

**Published:** 2024-01-08

**Authors:** Rachel Hird, Rajiv Radhakrishnan, Jack Tsai

**Affiliations:** ^1^Department of Psychiatry, Yale School of Medicine, New Haven, CT, United States; ^2^Department of Radiology and Biomedical Imaging, Yale School of Medicine, New Haven, CT, United States; ^3^Department of Veterans Affairs, National Center on Homelessness Among Veterans, Washington, DC, United States; ^4^School of Public Health, University of Texas Health Science Center at Houston, Houston, TX, United States

**Keywords:** Homelessness, psychiatric disorders, medication non-adherence, Housing First, Assertive Community Treatment, Long Acting Injectable Antipsychotic

## Abstract

**Introduction:**

Medication non-adherence is a significant problem among homeless individuals with psychiatric disorders in the United States. We conducted a systematic review to identify strategies to improve psychiatric medication adherence among homeless individuals with psychiatric disorders, including substance use disorders.

**Methods:**

We searched seven databases (MEDLINE, Embase, PsychInfo, Scopus, Web of Science, CDSR, and CENTRAL) and screened 664 studies by title and abstract followed by full-text review. Our inclusion criteria were studies that: involved an intervention for homeless adults with psychiatric disorders, reported a quantitative outcome of medication adherence, and were published in English in a peer-reviewed journal. We rated the relative effectiveness of strategies described in each study using a self-designed scale.

**Results:**

Eleven peer-reviewed studies met criteria for inclusion in this review. Within these studies, there were seven different approaches to improve medication adherence in this population. Three studies were randomized controlled trials (RCTs) and the remaining were observational studies. Outpatient interventions included Assertive Community Treatment, Cell Phone-Assisted Monitoring, Customized Adherence Enhancement plus Long-Acting Injectable Medications, and Homeless-Designated Pharmacy Clinics. Residential, shelter-based, and inpatient interventions included use of the Housing First model, Modified Therapeutic Communities, and Homeless-Designated Inpatient Care. The approaches described in four of the eleven studies were rated as scoring a 3 or higher on a 5-point scale of effectiveness in improving medication adherence; none received 5 points.

**Discussion:**

The interventions with the strongest evidence for improving medication adherence in this population were Assertive Community Treatment, Customized Adherence Enhancement plus Long-Acting Injectable Medications, and Housing First. Overall, studies on this topic required more rigor and focus on medication adherence as an outcome in this population. This review highlights several promising strategies and the need for larger RCTs to determine effective and diverse ways to improve medication adherence among homeless adults with psychiatric disorders.

## 1 Introduction

Medication non-adherence is an important problem among people with psychiatric disorders experiencing homelessness. Adherence to prescribed psychotropic drugs is associated not only with improved clinical outcomes but also improved housing outcomes (1). However, a minority (as low as 12%) of homeless individuals reach therapeutic efficacy with their prescribed psychotropics (2). Homelessness itself presents unique challenges to medication adherence, which require tailored approaches to improve adherence in this population. To our knowledge, there has been no systematic review of interventions targeted specifically to homeless adults with psychiatric disorders. In this paper, we present a systematic review of the literature on strategies that have been used to improve medication adherence among people with psychiatric disorders experiencing homelessness.

Homelessness is a recalcitrant public health problem in the United States that incurs high healthcare and societal costs. In the 2022 fiscal year, the U.S. allocated nearly $8 billion in federal funding for homeless assistance programs (3). Further, since 2021, the U.S. has allocated over $46 billion in emergency rental assistance to address the needs of an affordable housing crisis (4). Despite this, a significant proportion of persons with chronic homelessness continue to struggle with maintaining permanent housing. The U.S. Department of Housing and Urban Development (5) Annual Homeless Assessment Report to Congress notes that roughly 582,500 people were experiencing homelessness on a given night (5). That same year, 30% of those who experienced homelessness were chronically homeless, most of whom have psychiatric disorders (5). This marked an increase in chronic homelessness for the sixth year in a row, and the highest proportion of chronic homelessness reported in recent U.S. history (5).

Compared to housed individuals, homeless individuals report having a significantly lower quality of life in many domains, including safety, health, and social relationships (6). Compared to persons who were never homeless, those with unstable housing are more likely to be repeatedly hospitalized—including at residential/inpatient mental health facilities—and are also more likely to utilize acute care services, including urgent care and emergency departments (7–11). Homeless individuals also have a higher mortality rate than the general population (12–14), and this risk of mortality is particularly high in the United States compared to other developed countries (15). A significant proportion (30–40%) of chronically homeless people have a serious psychiatric disorder, such as schizophrenia and bipolar disorder (16, 17). Epidemiological and population-based studies in the United States estimate that 22–73% of homeless adults have a severe psychiatric disorder (7, 18–20); and conversely, 15% of people with a severe psychiatric disorder experience homelessness (7). Substance abuse and psychotic disorders have been identified as some of the strongest risk factors for homelessness aside from extreme poverty (7, 21, 22).

For chronically homeless individuals, one potential barrier to housing stability and improved quality of life is non-adherence to psychiatric medications. Antipsychotic medications are a mainstay first-line treatment for adults with schizophrenia-spectrum disorders and bipolar disorder (23, 24). While there remain concerns about side effects and variability in response to antipsychotic medications, rigorous large-scale studies have found that antipsychotic medications are effective in preventing symptom relapse and rehospitalization among adults with psychotic disorders (23, 25). Other psychotropic medications such as antidepressants and anxiolytics are also commonly used to treat mental health conditions among homeless adults (8, 26, 27). However, access to and adherence to psychotropic medications among homeless adults are of major public health concern (28, 29).

### 1.1 Prevalence of medication non-adherence among people with homelessness

As a result of a complex set of adherence challenges, medication non-adherence may be more prevalent among homeless individuals than housed individuals. Up to 60% of homeless individuals report having been prescribed a medication, while roughly one third report being unable to comply with dosing—particularly those who are younger or uninsured (9, 28, 30, 31). However, there is limited research that directly compares psychiatric treatment adherence between homeless and housed populations. Previous systematic reviews and retrospective studies of pharmacy records have found that psychiatric patients take on average 44–58% of their prescribed antipsychotics (32, 33), while homeless individuals take on average 30–41% of their prescribed antipsychotics (2, 34). An analysis of Medicaid claims and pharmacy records for individuals with schizophrenia in San Diego County, CA found that only 26% of the homeless population was adherent (medication possession ratio ≥ 0.8), whereas 36–50% of individuals in other living situations were adherent (35). Further, in a study of housed and homeless patients with HIV/AIDS, homeless individuals were significantly more likely to report having missed an antiretroviral dose in the past 48 h and having been noncompliant with their medication regimen in the past 30 days (36). Challenges with medication adherence for other diseases like tuberculosis and Hepatitis C have also been documented (37, 38). One previous national study, using administrative data from the U.S. Department of Veterans Affairs (VA) from 2010, examined the psychopharmacology of homeless veterans and found that homeless veterans with psychiatric disorders had 16% fewer psychotropic prescription fills than non-homeless veterans (8). This included 23% fewer antipsychotic refills and 25% fewer sedative-hypnotic refills. An analysis of VA National Psychosis Registry data found that among veterans with bipolar disorder and homelessness, only 38% reached the target adherence rate (80%) of their prescribed antipsychotic medication (39). In that sample, 62% of homeless veterans were non-adherent and 39% took less than half of their prescribed antipsychotics.

### 1.2 Factors contributing to medication non-adherence

Risk factors for psychiatric medication non-adherence among homeless people with psychiatric disorders include racial/ethnic minority background (40), major psychiatric disorders such as schizophrenia, bipolar disorder and substance use disorders, presence of cognitive impairment (41), and history of traumatic brain injury (42). There has not been adequate research to determine whether non-adherence to psychiatric medications is higher among homeless individuals with certain psychiatric conditions. A multivariable analysis of the 2003 Health Care for the Homeless User Survey found several factors independently associated with unmet needs for prescription medication, including lack of health insurance coverage, older age, out-of-home placement as a minor, past-year victimization, past-year employment, food insufficiency, and presence of two or more medical comorbidities (30). Employment may correlate with unmet healthcare needs because individuals living in poverty while employed (as opposed to receiving government benefits) are likely to be working jobs with unpredictable schedules, heavy consequences for absence, and no insurance benefits (30).

Individuals with homelessness must overcome a unique set of obstacles to adhere to a medication regimen. Challenges may include accessing a facility for regular refills, maintaining a reliable storage site for prescribed drugs, protecting drugs from theft, obtaining privacy for dosing, self-managing one’s doses, complying with medication instructions, and remaining engaged with treatment (9, 30, 31, 43, 44). Homeless individuals may not have regular daily schedules and reminders to take medications as prescribed; they may also have limited access to integrated care between their homeless service providers and prescribers; and many experience problems with substance use that may complicate their use and efficacy of psychotropic medications (2, 40, 45). Upon discharge from a psychiatric inpatient unit, homeless patients have less access than housed patients to critical healthcare resources, including case management services and prescription drug coverage, despite time of discharge being an ideal time for providers to arrange healthcare services for homeless patients (46). Nearly 60% of the U.S. homeless population is uninsured (30), which increases the financial burden of treatment and further decreases medication accessibility. A Canadian health care questionnaire found that among homeless men who did not fill a prescription medication, 73% reported non-adherence because of medication cost or lack of drug benefit coverage (43). Individuals who were automatically covered by a federal drug plan through their shelter were significantly less likely to leave prescriptions unfilled (20%, *N* = 20 vs. 6%, *N* = 6) (43). Homeless individuals in the United States similarly cite inability to afford care as the most common reason for unmet healthcare needs (30). This population may further limit healthcare encounters due to perceived discrimination in healthcare settings related to their homeless status (47). Homeless adults also self-report that poor self-management skills, lack of perceived effect, and forgetfulness are significant reasons for non-adherence to psychiatric medication (40). Lack of insight into one’s psychiatric condition and the importance of consistent treatment may also contribute to non-adherence. Lastly, mental health care may be neglected as homeless individuals are forced to prioritize more basic needs such as food, shelter, and safety (48).

### 1.3 Consequences of psychiatric medication non-adherence

The immediate consequence of medication non-adherence is that prescribed medications do not have the intended effects on patients’ health conditions. Consequences of non-adherence to psychiatric medications may include exacerbation or recurrence of psychiatric symptoms, further social or occupational impairments, and various other downstream outcomes such as hospitalization, financial instability, homelessness, and criminal justice involvement. Considerable literature has documented the negative consequences of psychiatric medication non-adherence in severe psychiatric conditions like schizophrenia and bipolar disorder (49–52). For example, data from the European Mania in Bipolar Longitudinal Evaluation of Medication (EMBLEM) study, which was a 21-month follow-up study, found that psychiatric medication non-adherence was significantly associated with increased risk of relapse, hospitalization, and suicide attempts (50). Costs incurred by non-adherent patients were significantly higher than those of adherent patients (£10,231 vs. £7,379) mainly due to inpatient costs. Another study estimated the annual inpatient costs of schizophrenia to be about $9 billion (adjusted for inflation) in the United States, with 40% of rehospitalization costs attributed to antipsychotic medication non-adherence (53).

Although there have been very few studies that have examined the consequences of psychiatric medication non-adherence specifically among homeless individuals, one would expect similar or worse consequences than those of stably housed individuals, given the potential negative downstream effects on housing and economic prospects. In one study of over 1,000 homeless or unstably housed adults in three Canadian cities, medication non-adherence was significantly associated with more frequent emergency department visits (three or more visits in a year) (28). Other Canadian studies have found that antipsychotic medication non-adherence is associated with longer lifetime duration of homelessness (2), while treatment adherence is associated with improved housing status as well as improved clinical outcomes for homeless individuals with psychiatric disorders (1). Poor health outcomes associated with psychiatric medication non-adherence may exacerbate the challenge of securing and maintaining housing, especially when the untreated illness involves cognitive impairment. Medication non-adherence is thus an important target for improving outcomes among homeless adults.

### 1.4 Assessing and reporting medication adherence

There are barriers to assessing the actual impact of interventions to improve medication adherence. Studies that measure adherence during a study may not be generalizable to real-world settings, and studies which rely on administrative records are reliant on documentation of medication adherence which may not always be captured. Further, among the existing research which focuses on medication compliance, “adherence” and “non-adherence” are defined differently between studies, which exacerbates the challenge of synthesizing the existing data. For example, patients are commonly considered “adherent” if they meet or exceed a certain threshold (commonly 80%) of prescribed doses (54, 55). However, studies may alternatively consider a patient “adherent” based on their regularity of dosing, e.g., the patient is adherent if they do not exceed some number of consecutive missed doses. Studies may use their own definition of adherence (sometimes unspecified) to report that a certain percentage of subjects were adherent, non-adherent, or partially adherent. Alternatively, adherence may be reported as a percentage of maximum possible engagement, e.g., a subject or group was 60% adherent if they attended 60% of scheduled treatment sessions. Thus, there is a lack of standardization in the reporting of medication adherence. There is also wide variability in the methods used to assess this adherence and the reliability thereof.

Medication adherence may be assessed using direct or indirect methods. Direct strategies are utilized less frequently because they involve more effort by the provider and the patient, and they are often more expensive than indirect methods (56). For instance, adherence can be monitored by plasma levels, although this is relatively burdensome and expensive. Direct monitoring of drug or drug metabolite concentration is also affected by “white coat adherence,” wherein adherence improves in the days before and after an appointment with a provider (57). Directly observed drug administration is the most reliable method of adherence monitoring, but also requires high effort (58). Prescription refill rate is one indirect measure that is widely used to assess adherence among psychiatric populations. Compared to other indirect measures of adherence, prescription refill rate is particularly accurate as it circumvents the Hawthorne effect, while data collection is relatively low effort and low cost (59). Pill counting is also popular, but not necessarily reliable, as pills may be discarded by the patient to give the illusion of adherence. Neither prescription refill rate nor pill counting validate when exactly each dose was taken or that it was taken at all (58). Dose timing can be observed via electronic pillbox monitoring, although this still does not confirm that the patient took the medication and dosed correctly. Electronic monitoring is also expensive to implement, which limits its current use (58). The most cost-effective and common method of assessing medication adherence is self-report by patients (56). However, self-reported adherence may be overestimated due to social desirability bias or recall issues, particularly among psychiatric patients with cognitive deficits (60). Still, the Medication Adherence Rating Scale (MARS) is popular for assessing medication adherence within psychiatric populations and has even been validated for use among homeless people with schizophrenia (61). The Modified Morisky Scale (MMS) has also been used to assess medication adherence among homeless individuals (62). The reliability of self-reported stigmatized behaviors (such as treatment noncompliance) may be improved by using computerized data collection rather than face-to-face interviewing (63).

To our knowledge, there has been no systematic review of interventions targeted specifically to homeless adults with psychiatric disorders. However, there is existing evidence that homeless individuals may be less compliant to interventions addressing medication adherence across conditions. A systematic review and meta-analysis of 771 interventions to address medication non-adherence, in general, across various populations found small effect sizes for interventions overall, and significantly lower effect sizes for interventions that included homeless populations compared to interventions that did not include homeless populations (0.160 vs. 0.292, respectively) (64). The review found that behavioral and habit-based interventions (e.g., rewards, prompts, linking dosing with another activity) were associated with higher adherence, whereas cognitive interventions (e.g., education, attitude improvement) were associated with lower adherence. It was also found that standardized interventions were more successful than individualized interventions. Of the 771 trials, only 17 reported including homeless individuals in the studied sample. The relative effectiveness of those 17 interventions was not reported. Further, psychiatric populations were not a focus of the review. The unique psychosocial, economic, and medical issues faced by homeless individuals with psychiatric conditions may require a more tailored approach to improving medication adherence. A synthesis of the literature may inform researchers and clinicians working with homeless populations and advise program administrators on effective ways to support homeless individuals in their recovery.

## 2 Methods

**Figure 1:**
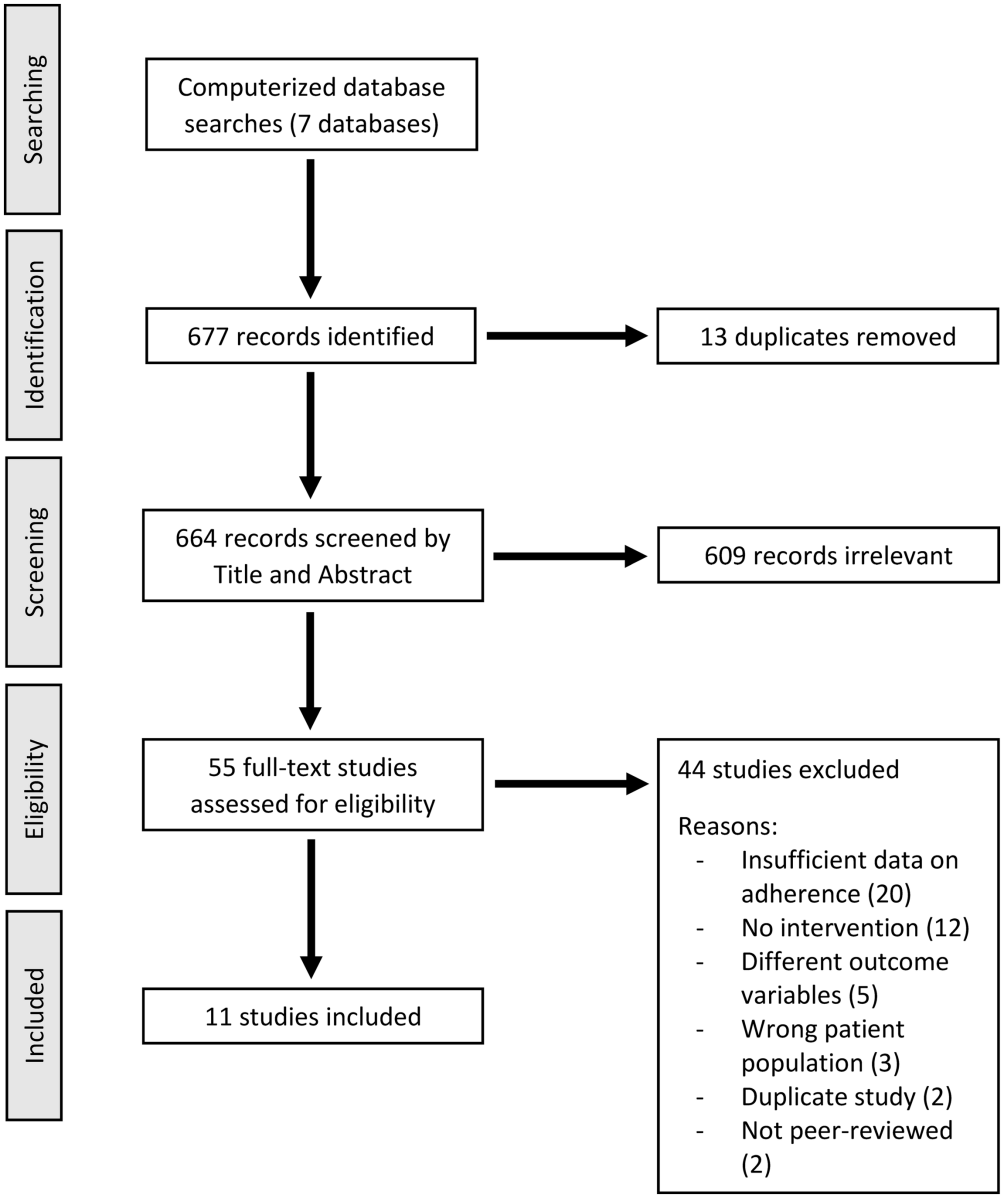
Flowchart of search strategy.

We conducted a systematic review of the literature on medication non-adherence among people with homelessness and psychiatric disorders with the assistance of the software Covidence. PRISMA guidelines for systematic reviews were followed, with some exceptions based on the quality of included studies and the specificity of available data (e.g., effect estimates were not calculated for each study). Databases searched included MEDLINE, Embase, PsychInfo, Scopus, Web of Science, CDSR, and CENTRAL. The search encompassed records up to August 11, 2022, with no lower limit. Search terminology included terms on homelessness, treatment adherence, and psychiatric disorders (see Supplementary Appendix 1). After excluding duplicate records, 664 studies were screened for the following criteria: (1) published in English, (2) published in a peer-reviewed journal, (3) including a healthcare intervention for adults with homelessness and psychiatric disorders, and (4) reporting quantitative data on psychiatric medication adherence such as: (a) pre- and post-intervention medication adherence, (b) between-group difference in medication adherence where groups receive different interventions, and (c) adherence level significantly different from established estimates for the population. Two authors screened titles and abstracts, and a third author resolved conflicts. After excluding 609 studies, two authors conducted a full-text assessment of 55 remaining studies using the same eligibility criteria. We identified 11 records that met criteria for inclusion. The search strategy is displayed in Figure 1.

We developed a scale consisting of five categories to rate the relative effectiveness of diverse strategies to improve medication adherence in this population as described in each study in the review. Each strategy that improved medication adherence was rated on a 5-point scale depending on whether it met each of the 5 items as reported in the study, which were: (1) At least 80% medication adherence was achieved; (2) Medication adherence improved by at least 50%; (3) Study was a randomized controlled trial; (4) Sample size was at least 30; and (5) Adherence was assessed at least 6 months post-intervention initiation. Studies which reported increased medication adherence and reported sufficient information to assess each of the five items were given a score out of five. Studies that did not report sufficient information to score one or more items were not assessed for relative effectiveness. The nature of items 1 and 2 (adherence level achieved and overall improvement) varied based on each study’s design and definition of adherence. For example, interventions could meet criteria for item 1 (“At least 80% adherence was achieved”) if at least 80% of the sample was deemed “adherent” post-intervention or if the average post-intervention medication possession ratio (MPR) was at least 0.8. Item 1 was incorporated because taking 80% of prescribed doses is a common threshold for medication therapeutic efficacy, and therefore it is a target adherence rate for individuals with homelessness and psychiatric disorders. When assessing item 2 (“Adherence improved by at least 50%”), improvement was calculated as a proportion of the sample’s baseline or control group adherence, e.g., if the sample’s mean MPR was 0.4 at baseline and 0.6 post-intervention, then adherence improved by 50%.

## 3 Results

This review identified a total of 11 studies which comprised seven different strategies to address medication non-adherence among people experiencing homelessness and psychiatric disorders (Table 1). Studies were conducted in the United States (eight interventions), Canada (two interventions), and England (one intervention). There were six studies in outpatient settings (four interventions), three studies in residential settings (two interventions), one study in a shelter setting (one intervention), and one study in an inpatient setting (one intervention). Of the 11 total studies, there were three randomized controlled trials (RCTs) and the remaining studies were observational studies. Across studies, sample sizes ranged from *N* = 10 to *N* = 165 (mean *N* = 75, median *N* = 52). Studies were published between 1997 and 2020. There was wide variability in adherence assessment method, definition of adherence, and reporting style for adherence data. The strategies used to improve medication adherence can be broadly divided based on location of care into (A) outpatient treatment strategies and (B) residential/shelter-based/inpatient treatment strategies.

**Table 1:** Strategies to address medication non-adherence among people with homelessness and psychiatric disorders.

Intervention	Study	Setting	Study design	Comparison	Psychiatric population	Sample size	Duration of intervention	Adherence assessment method	Results	Description of results	Quality of study (score on 5-Item Scale)
Assertive Community Treatment	(65)	Outpatient	Randomized controlled study	Within-subjects	Severe and persistent mental illness	77^1^	1 year	Psychiatrists’ assessment	Increased medication adherence	Proportion of clients adherent increased from 29% at baseline to 57% at 3 months	Between-subjects adherence data was not reported (Score = 4)
Cell Phone-Assisted Monitoring	(66)	Outpatient	Non-randomized uncontrolled study	None	Comorbid Axis I and substance use disorder	10	1 month	Self-report	Increased medication adherence likely	93% of doses were taken	No comparison group or baseline data; Results rely on self-report; Adherence was measured short-term (30 days) (Score = N/A)
Customized Adherence Enhancement plus Long-Acting Injectable Antipsychotics	(67)	Outpatient	Non-randomized uncontrolled study	Within-subjects	Schizophrenia or schizoaffective disorder	30^2^	6 months	Self-report (Tablets Routine Questionnaire)	Increased medication adherence	Mean missed doses decreased from 46% at enrollment to 10% at 6 months	Results rely on self-report (Score = 3)
Customized Adherence Enhancement plus Long-Acting Injectable Antipsychotics	(68)	Outpatient	Non-randomized uncontrolled study	Within-subjects	Schizophrenia or schizoaffective disorder	30^3^	6 months	Self-report (Tablets Routine Questionnaire)	Increased medication adherence	Mean missed doses decreased from 49% at enrollment to 15% at 6 months	Results rely on self-report (Score = 3)
Homeless-Designated Pharmacy Clinic	(69)	Outpatient	Non-randomized uncontrolled study	Within-subjects	Veterans prescribed psychotropic medications	52^4^	1–2 visits (each 5–30 min)	Medication possession ratio	Increased medication adherence	Mean medication possession ratio increased from 46.6 to 60.7% at 30 days pre- vs. post- intervention	Adherence was measured short-term (30 days post-intervention) (Score = 0)
Homeless-Designated Pharmacy Clinic	(70)	Outpatient	Non-randomized uncontrolled study	Within-subjects	Veterans prescribed mental health medications	21	1–2 visits (each 30 min)	Not specified	Increased medication adherence	Of 18 veterans with noncompliance, 5 (28%) improved adherence	Adherence assessment method was not specified; Degree of improvement was not specified; Timepoint of assessment was not specified (Score = N/A)
Housing First with Assertive Community Treatment or Other Health and Social Services	(71)	Residential	Randomized controlled study	Between-subjects	Opioid dependence and mental illness	97	2.8 years	Medication possession ratio	No difference in medication adherence	No significant difference in mean medication possession ratio	(Score = N/A)
Housing First with Assertive Community Treatment or Other Health and Social Services	(72)	Residential	Randomized controlled study	Between-subjects	Schizophrenia	165	2.6 years	Medication possession ratio	Increased medication adherence	Higher medication possession ratio in scattered-site housing with Assertive Community Treatment (0.78) compared to congregate housing (0.61) and control group (0.55)	(Score = 3)
Therapeutic Community, Modified for Homelessness Prevention	(73)	Residential	Non-randomized, non-equivalent controlled study	Between-subjects	Mothers with substance abuse	148^5^	1 year	Not specified	No difference in medication adherence	No significant difference in medication adherence between modified and standard Therapeutic Communities	Respective levels of adherence were not specified; Control was standard Therapeutic Community, not treatment as usual
Therapeutic Community, Modified for Homeless Persons with Co-occurring Disorders	(74)	Shelter-based	Retrospective controlled study	Between-subjects	Comorbid substance use disorders and mental illness	140	8.3 months (mean for veteran subset)	Case records	Increased medication adherence	Lower proportion of subjects nonadherent in Modified Therapeutic Community (18.6%) vs. control group (35.3%)	Definitions of adherence, partial adherence, and non-adherence were not specified (Score = 2)
Homeless-Designated Inpatient Facility	(75)	Inpatient	Non-randomized controlled study	Between-subjects	Mental illness	50^6^	5.8 months (mean)	Care coordinators’ assessment (Rating of Medication Influences)	Increased medication adherence likely	Higher proportion of experimental group improved medication noncompliance influences (95% vs. 46%)	Assessed medication adherence indirectly (Score = N/A)

^1^*N* = 72 subjects assessed for medication adherence. ^2^*N* = 10 subjects assessed for concomitant oral medication adherence. ^3^*N* = 15 subjects assessed for concomitant oral medication adherence. ^4^*N* = 17 subjects assessed for medication adherence. ^5^*N* = 49 subjects assessed for medication adherence. ^6^*N* = 32 subjects assessed for medication adherence.

Nine of eleven studies reported increased adherence. Positive adherence outcomes were associated with all four outpatient interventions, including: Assertive Community Treatment (ACT), Cell Phone-Assisted Monitoring, Customized Adherence Enhancement (CAE) plus Long-Acting Injectable (LAI) Medications, and Homeless-Designated Pharmacy Clinics. Two outpatient interventions, CAE plus LAI Medications and Homeless-Designated Pharmacy Clinics, were each supported by two studies. The two other outpatient interventions were each examined in one study. Among residential, shelter-based, and inpatient strategies, mixed adherence outcomes were associated with two out of three interventions: use of the Housing First model and Modified Therapeutic Communities. Each of these interventions was examined in two studies. Positive adherence outcomes were associated with a Homeless-Designated Inpatient Facility, which was examined in one study.

### 3.1 Outpatient treatment strategies

#### 3.1.1 Assertive Community Treatment

One RCT study of Assertive Community Treatment (ACT) (65) met the inclusion criteria of this review. The ACT model is a strong evidence-based model of care for adults with psychiatric disorders including those experiencing homelessness (76–78). Clients are engaged in team-based treatment, which is focused on helping clients to (1) acquire material resources (food, shelter, etc.); (2) develop community-life coping skills (using public transport, budgeting money, etc.); (3) remain motivated to persevere; and (4) develop greater autonomy (77). ACT also involves supporting and educating non-patient community members to better relate to patients. All aspects of this treatment model are “assertively” promoted to minimize dropout. Program administrators and evaluators have reported increased levels of medication adherence among homeless people with psychiatric disorders engaged in ACT (79). Dixon et al. examined medication adherence among homeless individuals (*N* = 77) with severe and persistent psychiatric disorders (schizophrenia, major affective disorder, or primary substance use disorder) engaged in ACT or usual community services (65). In the experimental group, percentage of patients who were medication adherent nearly doubled (from 29 to 57%) between baseline and 3 months of ACT. Adherence remained similarly high 1 year after baseline (65). Subjects were deemed non-adherent, intermittently adherent, or adherent at each three-month evaluation point. If subjects missed doses for more than seven consecutive days or refused psychotropic medication suggested by a psychiatrist, they were deemed non-adherent. Frequency of non-consecutive missed doses was also taken into account. Program psychiatrists used a variety of factors to assess for non-adherence, including hospital records, pill counts, blood levels, reports from the patient, and input from their clinicians, family members, and community supports.

#### 3.1.2 Cell Phone-Assisted Monitoring

One non-randomized uncontrolled pilot study on Cell Phone-Assisted Monitoring of medication adherence (66) met inclusion criteria. An automated, cell phone-based medication monitoring system was identified as a feasible method of monitoring psychiatric medication adherence for homeless patients (66). Ten homeless individuals with comorbid psychiatric disorders and substance abuse were enrolled in a 30-day pilot study wherein they received automated daily phone calls to assess medication adherence (66). Participants were reachable 93% of the time and self-reported 100% adherence when reached. Baseline adherence was not measured, and adherence was not verified with additional methods. Over the 30-day trial, all phones were retained by participants and there were no dropouts. Upon study exit, participants reported that the automated system reminded them to medicate and added structure to their day (66).

#### 3.1.3 Customized Adherence Enhancement plus Long-Acting Injectable Antipsychotics

This review identified two studies which implemented a long-acting injectable (LAI) intervention for homeless individuals, both of which combined LAI antipsychotics with Customized Adherence Enhancement (CAE) and utilized a non-randomized uncontrolled study design (67, 68). An earlier study found that switching veterans from oral to LAI antipsychotics was associated with fewer inpatient psychiatric admissions and shorter inpatient stays (80). Sajatovic et al. found that concomitant LAI antipsychotic treatment with haloperidol decanoate resulted in increased adherence to oral non-antipsychotic psychotropic medications after 6 months among homeless individuals with psychotic disorders (*N* = 30) (67). Participants in this study received monthly CAE in addition to the monthly LAI. CAE included medication-related psychoeducation (developing medication routines, communicating medication burdens with providers, managing adherence, etc.). In a subset of 10 subjects who were prescribed non-antipsychotic oral psychotropic medications, missed doses (past month) of prescribed oral psychotropics decreased from 46.1% at study enrollment to 10.1% at study end. Missed doses were assessed by self-report (modified Tablets Routine Questionnaire). The combined CAE and LAI treatment also improved psychiatric symptoms and functioning in the homeless adults studied (67). A second six-month CAE plus LAI antipsychotic study with similar structure found that concomitant LAI paliperidone palmitate improved adherence to oral prescribed drugs among homeless individuals with psychotic disorders (*N* = 30) (68). In a subset of 15 subjects, missed doses (past month) of oral prescribed drugs decreased from 48.7% at enrollment to 15.2% at study end based on self-report (Tablets Routine Questionnaire) (68).

#### 3.1.4 Homeless-Designated Pharmacy Clinics

Two Homeless-Designated Pharmacy Clinic interventions were included in this review, and both used a non-randomized uncontrolled study design (69, 70). The US Department of Veterans Affairs has created a Homeless Patient Aligned Care Team (H-PACT), a treatment model to help provide primary care to homeless veterans (81). In one study, a pharmacy resident clinic was established at a day center for homeless veterans, partially to support the need for local H-PACT implementation (69). This walk-in clinic was open one half-day per week. A psychiatric pharmacy resident met with veterans to review medications, provide medication counseling and other education, discuss patient concerns, and implement related interventions, among other services. Visits lasted 5–30 min. Over 18 clinic days, 52 veterans attended the clinic and 17 of those veterans were prescribed psychotropic medications. Following engagement with the clinic, average psychotropic medication adherence increased from 46.6 to 60.7%. Adherence was assessed by MPR 30 days prior to and 30 days after the veteran’s pharmacy clinic visit. A second study described adding a mental health pharmacy resident clinic within H-PACT at one location to improve mental health access for its patients (70). The pharmacy resident clinic evaluated veterans during 30-min in-person visits. Veterans were provided with medication adherence education as well as other medication-related services (reduction in polypharmacy, identifying administration errors, regimen adjustments, referrals, etc.). In total, 21 veterans received pharmacotherapy assessment at the clinic, 18 were noncompliant to some extent, and 5 improved adherence following service engagement. The study’s assessment strategy for adherence was not specified, nor was the degree of improvement or the timepoint of follow-up.

### 3.2 Residential/shelter-based/inpatient treatment strategies

#### 3.2.1 Housing first

Two randomized controlled Housing First studies met the inclusion criteria of this review, with mixed results for adherence improvement (71, 72). Given the varied challenges faced by homeless individuals receiving psychiatric treatment, Housing First is a prominent strategy to improving outcomes (82, 83). When the basic need of stable housing is secured, patients may prioritize secondary needs like psychiatric treatment. In this model, housing is not contingent on treatment or abstinence. One study assigned opioid-dependent homeless adults with psychiatric disorders (*N* = 97) to Housing First or treatment-as-usual and found that Housing First did not increase adherence to methadone maintenance treatment (71). Housing First group participants were assigned to one of three types of housing based on need assessment, including (1) participant’s choice of market rental apartment plus ACT, (2) participant’s choice of market rental apartment plus intensive case management with referrals to community services, and (3) study-specific building with private living quarters, some shared amenities (kitchen and dining room), and 24/7 on-site health service providers. Difference in adherence between the three Housing First groups, if any, was not reported. Adherence was based on MPR, which was calculated from methadone dispensation data. In the post-randomization period, mean MPR was 0.52 for Housing First and 0.57 for controls, with no statistically significant between-subjects difference. A second Housing First RCT study found that Housing First increased adherence to antipsychotics among formerly homeless individuals with schizophrenia (*N* = 165) when randomized to scattered-site market rentals with ACT (72). In a randomized controlled trial, participants were assigned to treatment-as-usual or one of two Housing First groups: congregate Housing First wherein clients were assigned single-occupancy units in a shared building with on-site supports or scattered-site Housing First wherein clients chose a single-occupancy market rental and were engaged in ACT. MPR was used to assess adherence. The congregate Housing First group exhibited very low adherence in the post-randomization period (mean MPR 0.61), with levels similar to the treatment-as-usual group (mean MPR 0.55). Significantly higher antipsychotic medication adherence was observed in the scattered-site Housing First plus ACT group (mean MPR 0.78) (72).

#### 3.2.2 Therapeutic communities

One non-randomized non-equivalent controlled study on Modified Therapeutic Community (73) met criteria for inclusion. The Therapeutic Community model, originally developed for the treatment of substance abuse, facilitates overall lifestyle changes (psychological, medical, social, legal, etc.) in support of recovery (74). Residential Therapeutic Communities have been shown to decrease substance use and improve psychological functioning. Many modifications of the Therapeutic Community exist to serve different patient populations. One study examined the effects of a Modified Therapeutic Community (MTC) for homeless mothers with substance abuse in comparison to a standard Therapeutic Community (73). Modifications in the experimental MTC program addressed needs related to family stabilization and homelessness prevention. The experimental group (*N* = 77) included two MTC programs and the control group (*N* = 71) included two standard residential Therapeutic Community programs, with statistical control adjusting for between-group differences. Medication adherence was assessed as part of a greater “Health” domain for each participant, which also included self-help group attendance and amount of help received in understanding medications. Eight of ten items in the “Health” domain were improved in the experimental MTC group, but the exact difference in self-help group attendance and medication adherence was not specified, and the between-group difference in these factors was not statistically significant (73).

A second Modified Therapeutic Community study used a retrospective controlled design (74). The study investigated the effects of a shelter-based Therapeutic Community, modified to address the needs of homeless people with co-occurring substance use disorders and psychiatric disorders (74). Modifications included shortening the duration of activities and meetings, presenting clinical information in smaller units with increased discussion, more hands-on assistance from staff, and more individual counseling. Emphasis was placed on understanding one’s psychiatric illness and avoiding relapse triggers. The quasi-experimental study utilized a comparison group of homeless veterans with co-occurring disorders in a general shelter without Therapeutic Community. The MTC group was mostly comprised of non-veterans, with a subset of veterans. Each subject was deemed adherent, partially adherent, or non-adherent, although we were unable to determine exactly how the study defined each. Based on a retrospective review, the control group was significantly more non-adherent, with 35.3% of residents no-adherent in the general shelter without Therapeutic Community (*N* = 70) and 18.6% non-adherent in the MTC shelter (*N* = 70) (74). Adherence and partial adherence were reported only for the veteran subset. The proportion of veterans fully and partially adherent was higher in the experimental MTC than in the control group (60.0% fully adherent and 28.0% partially adherent in the MTC vs. 55.9% fully adherent and 8.8% partially adherent in the control group) (74).

#### 3.2.3 Homeless-Designated Inpatient Facility

One non-randomized controlled study examining the effectiveness of a Homeless-Designated Inpatient Facility (75) met inclusion criteria for this review. Previous research has demonstrated that patients with schizophrenia improve medication adherence following inpatient hospitalization (84). One study evaluated the effect of admission to a homeless-designated inpatient ward on various outcome measures among homeless patients with psychiatric disorders (75). Control patients (*N* = 21) were admitted to standard inpatient units, while the experimental group (*N* = 29) was admitted to a homeless-designated unit that also included enhanced coordination of discharge planning (75). Average length of stay was 177 days for the experimental group and 105 days for the control group. At baseline, factors influencing medication adherence were evaluated using the Rating of Medication Influences (ROMI) (85), which identifies influencing factors of medication compliance and noncompliance separately. Ratings were repeated by care coordinators at 12 months post-discharge for 32 participants. Experimental group participants were more likely to demonstrate improvement on medication noncompliance influences (95% of experimental group vs. 46% of control group) (75). The groups were equally likely to improve on compliance influences. Medication adherence was not measured directly in this study.

### 3.3 Rating of effectiveness of different strategies

Using our 5-item scale of effectiveness of strategies to improve medication adherence in this population, we scored the studies included in this review (Table 1). However, three of the nine studies in the review which reported increased medication adherence were missing information for one or more items so they could not be scored. The Cell Phone-Assisted Monitoring pilot study (66) reported the highest medication adherence rate (mean 93%) but did not utilize a comparison group or measure baseline adherence to assess improvement. One Homeless-Designated Pharmacy Clinic study (70) reported that 28% of clients improved medication adherence but did not specify the nature of this improvement. The Homeless-Designated Inpatient Facility intervention utilized scales related to medication adherence, but adherence itself was not measured.

## 4 Discussion

Medication non-adherence is a well-documented and widely known problem among people experiencing homelessness and psychiatric disorders. The effectiveness of interventions targeting medication adherence in this population has not been systematically examined to date. The results of this systematic review show that effective, RCT-supported strategies to improve medication adherence among homeless individuals with psychiatric disorders include Assertive Community Treatment (ACT) and Housing First. Non-RCT studies support the effectiveness of long-acting injectable antipsychotics combined with Customized Adherence Enhancement (CAE plus LAI), Therapeutic Community, and Homeless-Designated Pharmacy Clinics, although further validation in RCTs is warranted.

Of the nine interventions with positive adherence outcomes, ACT was the most effective intervention. After 3 months of ACT, the proportion of homeless subjects with psychiatric disorders who were adherent to medication increased substantially (from 29% adherent at baseline to 57% adherent at 3 months) and remained at a similar level 1 year after baseline (65). This study was an RCT that assessed medication adherence in 72 subjects. A previous meta-analysis (86) reported that ACT reduces homelessness and psychiatric symptom severity in individuals experiencing homelessness and psychiatric disorders; positive outcomes may be due in part to increased psychiatric medication adherence. It is important to recognize that some critics have pointed to ACT being coercive or too “paternal,” but there are ways to structure ACT and to build a team culture that is recovery-oriented in serving homeless populations (87). In addition, Housing First interventions, which were included in this review and are client-centered, often use an ACT-like model for case management and demonstrate how these models might be effectively used within a recovery-oriented framework.

Long-acting injectable antipsychotics combined with Customized Adherence Enhancement (CAE plus LAI) (67, 68) achieved high rates of adherence (mean 89.9 and 84.8% of doses were taken, respectively) at 6 months post-intervention initiation. LAI antipsychotic medications were initiated as a component of these interventions; the reported adherence improvements refer to concomitant oral psychotropic medications. CAE plus LAI Medication appears to be a promising strategy to improve oral psychiatric medication adherence among homeless individuals with schizophrenia. Future studies would benefit from larger sample sizes and RCT design.

A Housing First intervention (72) was associated with significant improvement in adherence (mean MPR 0.78, 41.8% improvement in adherence). The study included a scattered-site housing group, which had higher medication adherence than the congregate housing group. ACT was incorporated into the scattered-site Housing First intervention. The study was a well-powered RCT with an adequate sample size (*N* = 165) and a long-term adherence measurement (mean follow-up time was 2.6 years). Providing homeless individuals with supported housing may be a promising strategy to improve medication adherence. But a second Housing First RCT study in this review (71) which used a smaller sample size did not report significantly improved medication adherence. The two Housing First studies differed in psychiatric population and the associated medication type on which adherence was based. Improved adherence was reported for homeless adults with schizophrenia taking antipsychotic medication (72), but adherence improvement was not reported for homeless adults with opioid dependence and psychiatric disorders taking methadone (71). The additional challenges faced by dual-diagnosed homeless individuals may contribute to smaller improvement from interventions like Housing First. Other factors that may contribute to adherence improvement are the type of housing provided (e.g., market rentals or program-specific housing), the desirability thereof, and the intensity of bundled health services. The provision of market-based, single-occupancy apartments was associated with improved medication adherence whereas more communal housing (a study-designated building with single-occupancy rooms, communal meals, and on-site supports) was not associated with improved adherence despite there being no significant difference in demographics or pre-randomization adherence between groups (72). A previous multisite study analysis found that homeless clients in substance abuse treatment significantly increase retention when housing is provided, but that retention may suffer when housing is provided alongside less desirable high-intensity services (88). It may be useful to examine adherence improvement among homeless adults with psychiatric disorders when Housing First is combined with supportive services of different intensities.

Non-RCT studies focused on the homeless population with psychiatric disorders include Therapeutic Community and Homeless-Designated Pharmacy Clinics. A Therapeutic Community modified for homeless individuals with comorbid substance use disorders and psychiatric disorders (74) reported modest improvement in adherence compared to other interventions (25.8% improvement in proportion of sample adherent and partially adherent). The study used a retrospective controlled design, although it was of an adequate sample size (*N* = 140) and included long-term assessment of medication adherence (mean length of stay was 8.3 months in the veteran subset). A Homeless-Designated Pharmacy Clinic study (69) reported that mean adherence improved from 46.6 to 60.7% in a small subset of veterans (*N* = 17). A non-RCT design was used, and adherence was measured 30 days post-intervention. This pharmacy clinic was the shortest intervention to report significant adherence improvement, with clinic visits lasting a maximum of 30 min and most veterans attending only one visit.

RCTs of interventions involving telehealth and incentivized programs to increase medication adherence are needed. Given the increasing utilization of telehealth services and patients’ increasing comfort with mobile devices, cell phone-assisted medication adherence strategies warrant further exploration. Forgetfulness as a factor of non-adherence may be reduced by automated reminders. Further, habit-based and behavioral-focused interventions have shown to be more successful at improving adherence than cognitive-based interventions in the general population (64); this finding may be extended to homeless individuals with psychiatric disorders, who may especially benefit from increased daily regimentation. To implement a telehealth intervention, homeless patients must be provided with mobile devices and a service plan, but the strategy otherwise requires relatively little ongoing effort or financial investment. While electronically self-reported adherence may be exaggerated, patients may also be more honest about stigmatized behaviors (e.g., medication noncompliance) with computerized systems than with providers in-person (63). This review included one pilot telehealth study on Cell Phone-Assisted Monitoring of daily medication adherence (66) wherein subjects retained the provided cell phones, appreciated that automated calls added structure to their day, and reported very high medication adherence (mean 93% of doses were taken). Further study is necessary to determine the effectiveness of Cell Phone-Assisted Monitoring in this population long-term. It would be useful to validate electronically self-reported adherence using pharmacy records or other methods. Further study is also needed on interventions utilizing financial incentives to increase medication adherence among homeless adults with psychiatric disorders. A previous meta-analysis (89) found that incentivized programs significantly increase medication adherence in individuals with psychiatric disorders, and a previous scoping review (90) found that financial incentives may improve engagement and retention in health services for homeless adults. This review did not include incentivized interventions. Also conspicuous by their absence are studies that incorporate cognitive remediation and vocational rehabilitation strategies.

The conclusions of our systematic review need to be viewed in the context of its limitations. This review focuses on challenges surrounding homelessness primarily in the United States, and nine of twelve studies included in this review were conducted in the United States. Further, there is a very limited number of studies which fit the search criteria of this review. Medication adherence was often not the primary outcome variable in these studies, so adherence data was accompanied by minimal or no statistical analysis in many cases. We also found that in studies reporting medication adherence following an intervention, baseline adherence was often not assessed or not reported. Given the small number of studies in this review, we were also hesitant to distinguish between medication-assisted addiction treatments (e.g., opioid replacement therapy) and other psychotropic medications. The strategies best suited to enhancing medication adherence may vary based on the psychiatric condition under treatment and the presence of comorbidities. Among the studies included in this review, medication adherence was measured using different strategies and reported with varying levels of specificity. As a result, it is challenging to directly compare the effectiveness of these interventions, such as through a meta-analysis. More research is needed on additional strategies to improve medication adherence in this population, including telehealth and incentivized programs. It would also be beneficial to analyze existing strategies separately, as they are often combined for therapeutic effect, e.g., Customized Adherence Enhancement plus Long-Acting Injectable Medication. Among the existing intervention studies targeting medication adherence in people with psychiatric disorders experiencing homelessness, there are few randomized controlled trials.

## 5 Conclusion

Among the interventions included in this systematic review, the interventions with the strongest evidence for improving medication adherence among individuals with psychiatric disorders experiencing homelessness were Assertive Community Treatment, Customized Adherence Enhancement plus Long-Acting Injectable Medication, and Housing First. Smaller, non-randomized, and/or uncontrolled trials of Cell Phone-Assisted Monitoring, Homeless-Designated Pharmacy Clinics, Therapeutic Community, and Homeless-Designated Inpatient Care also showed improved adherence. Given the importance of medication adherence in this population, additional adequately powered randomized controlled trials examining medication adherence improvement strategies are warranted.

## Data availability statement

The original contributions presented in the study are included in the article/Supplementary material, further inquiries can be directed to the corresponding author.

## Author contributions

RH: Writing – original draft, Writing – review & editing. RR: Writing – original draft, Writing – review & editing. JT: Writing – original draft, Writing – review & editing.
