# Supplementary material for: A systematic review of approaches to improve medication adherence in homeless adults with psychiatric disorders
Source: Front Psychiatry. 2024 Jan 8;14:1339801. doi: 10.3389/fpsyt.2023.1339801 (PMC10800888; doi:10.3389/fpsyt.2023.1339801)
Supplement: Supplementary file 1 [file Table_2.pdf]

Supplementary Appendix 1
Database search terminology.

1	exp homeless persons/ or (Housing instabilit* or Homeless* or Unhoused or unstable housing or un housed or unhoused or run away* or runaway* or (street adj3 (youth* or child* or people))).mp. or (houseless* or vagrant* or vagrancy or unsheltered or "people living on the street*" or ((sleep* or living) adj3 rough)).mp.
2	Medication adherence/ or ((Medication or treatment or drug or dose or dosing or dosage) adj6 (Non-adheren* or Non-complian* or adheren* or nonadheren* or noncomplian* or complian* or persisten*)).mp.
3	1 and 2
4	mental disorders/ or depression/ or Anxiety/ or exp Anxiety Disorders/ or exp Depressive Disorder/ or biopolar disorder/ or exp "schizophrenia spectrum and other psychotic disorders"/ or substance-related disorders/ or exp "trauma and stressor related disorders"/
5	(mental health or mentally ill or mental illness or psychiatric disorder* or psychiatric illness* or behavioral health or behavioural health or mental disorder* or mental disease* or bipolar or schizophren* or psychotic or psychosis or psychoses or "substance use" or substance related or substance misuse or post traumatic or PTSD or alcohol* or cocaine or opioid* or opiate*).mp.
6	4 or 5
7[a]	3 and 6

[a] Final search terminology
